# A Higher Fructose Intake Is Associated with Greater Albuminuria in Subjects with Type 2 Diabetes Mellitus

**DOI:** 10.1155/2018/5459439

**Published:** 2018-10-17

**Authors:** Miguel Ángel Gómez-Sámano, Paloma Almeda-Valdes, Daniel Cuevas-Ramos, María Fernanda Navarro-Flores, Héctor Donaldo Espinosa-Salazar, Mayela Martínez-Saavedra, Jefsi Argelia León-Domínguez, Víctor Manuel Enríquez-Estrada, Ana Laura López-González, Ana Laura Sarmiento-Moreno, Lucero Alejandra Rivera-González, Óscar Alfredo Juárez-León, Bernardo Pérez-González, Yessica Ávila-Palacios, Lineth Sigala-Pedroza, Eira Huerta-Ávila, María Angelina Vargas-Álvarez, Carlos Sánchez-Jaimes, Mariana Cárdenas-Vera, Roopa Mehta, Manuel Alejandro López-Flores A La Torre, Iliana Manjarrez-Martínez, Griselda Xochitl Brito-Córdova, Julia M. Zuarth-Vázquez, Arturo Vega-Beyhart, Guadalupe López-Carrasco, Richard J. Johnson, Francisco Javier Gómez-Pérez

**Affiliations:** ^1^Department of Endocrinology and Metabolism, Instituto Nacional de Ciencias Medicas y Nutricion Salvador Zubiran, 14080 Mexico City, Mexico; ^2^Division of Renal Diseases and Hypertension, University of Colorado, Denver 12631, Aurora, USA

## Abstract

The aim of this single center cross-sectional study was to investigate the association between fructose intake and albuminuria in subjects with type 2 diabetes mellitus (T2DM). This is a single center cross-sectional study. One hundred and forty-three subjects with T2DM were recruited from the Instituto Nacional de Ciencias Medicas y Nutricion Salvador Zubiran. The median daily fructose intake was estimated with a prospective food registry during 3 days (2 week-days and one weekend day) and they were divided into low fructose intake (<25 g/day) and high fructose intake (≥ 25 g/day). Complete clinical and biochemical evaluations were performed, including anthropometric variables and a 24-hour urine collection for albuminuria determination. One hundred and thirty-six subjects were analyzed in this study. We found a positive significant association between daily fructose intake and albuminuria (*ρ*= 0.178, p=0.038) in subjects with type 2 diabetes mellitus. Other variables significantly associated with albuminuria were body mass index (BMI) (*ρ*= 0.170, p=0.048), mean arterial pressure (MAP) (*ρ*= 0.280, p=0.001), glycated hemoglobin (A1c) (*ρ*= 0.197, p=0.022), and triglycerides (*ρ*= 0.219, p=0.010). After adjustment for confounding variables we found a significant and independent association between fructose intake and albuminuria (*β*= 13.96, p=0.006). We found a statistically significant higher albuminuria (60.8 [12.8-228.5] versus 232.2 [27.2-1273.0] mg/day, p 0.002), glycated hemoglobin (8.6±1.61 versus 9.6±2.1 %), p= 0.003, and uric acid (6.27±1.8 versus 7.2±1.5 mg/dL), p=0.012, in the group of high fructose intake versus the group with low fructose intake, and a statistically significant lower creatinine clearance (76.5±30.98 mL/min versus 94.9±36.8, p=0.014) in the group with high fructose intake versus the group with low fructose intake. In summary we found that a higher fructose intake is associated with greater albuminuria in subjects with T2DM.

## 1. Introduction

Fructose is a monosaccharide that is present in significant amounts in fruits, honey, table sugar (sucrose has a 50% fructose content), and soft drinks sweetened with high fructose corn syrup (55% fructose content) [1]. It is the sweetest and more soluble natural carbohydrate [2].

Due to its low cost [3], it was adopted by the soft drink industry, and nowadays many sugar-sweetened beverages and fruit juice drinks contain high amounts of fructose [4].

To give an example of the current data about fructose added beverages intake, sugar-sweetened soft drink consumption increased 400% in Brazilian metropolitan areas from 1974–1975 to 2002–2003, corresponding to an increase of 0.4% to 2.1% of the total calories consumed daily [5]. In the Institute of Medicine (IOM) macronutrient report, the committee recommended a maximal intake of ≤ 25% of energy from added sugars. In a recent survey thirteen percent of the population had added sugars intake > 25% [6].

The intake of sweetened beverages high in fructose increased by >1000% between 1970 and 1990 [7]. In 2013, 68% of all packaged foods and beverages purchased by a nationally representative sample of United States households contained caloric sweeteners [8].

Unlike glucose that has a glycemic index of 59, fructose has a lower glycemic index of 20 [2]. In addition, it induces less insulin secretion compared to glucose [9]. Because of these characteristics, fructose has been recommended as a substitute for glucose in patients with diabetes [10, 11].

However, this recommendation has been challenged by experimental and epidemiological evidence that excessive fructose intake may have a role in the development of insulin resistance, metabolic syndrome, and type 2 diabetes mellitus (T2DM) [12, 13]. Currently, it is well known that high fructose intake favors the development and progression of kidney disease [14]. For example, rats fed with a high fructose diet developed tubular injury and interstitial inflammation and fibrosis, associated with an increased oxidative stress [15]. A similar study showed that a high fructose diet accelerated the progression of renal damage, causing glomerular sclerosis, cellular infiltration, tubular dilatation, and atrophy [16]. An association between the intake of more than two sugary soft drinks per day and prevalence of albuminuria was also observed in a study analyzing data from the National Health and Nutrition Examination Survey (NHANES) [17].

The primary aim of this study was to evaluate the association between fructose intake and albuminuria. The secondary aims were to evaluate the association between fructose intake and the following: uric acid, glycemic control, creatinine clearance, lipid profile, and anthropometric variables, in Mexican-Mestizo subjects with T2DM. We hypothesized that in subjects with T2DM, increased fructose intake is associated with greater albuminuria; our secondary hypothesis was that a higher fructose intake is associated with a higher uric acid serum concentration, lower creatinine clearance, higher triglycerides, and higher BMI.

## 2. Material and Methods

This was a single center cross-sectional study. Subjects were recruited from the Diabetes Clinic at the Instituto Nacional de Ciencias Medicas y Nutricion Salvador Zubiran, a nonprofit, public, and university-affiliated hospital in Mexico City, between June 2013 and March 2015. We included subjects of both genders between 18 and 70 years old, with T2DM, with a median of 16 [3–36] years of duration of diabetes, and albuminuria between 0 and 6000 mg/day determined in the prior three months. If the subjects had albuminuria ≥30 mg/day, they needed to have documented diabetic retinopathy to confirm that nephropathy is due to diabetes mellitus 2 and be able to complete the prospective diary of food ingestion. All subjects who presented albuminuria determination within 3 months of enrollment were invited to participate. This albuminuria determination was used as the outcome variable in our statistical analysis. Exclusion criteria included a body mass index (BMI) >40 kg/m^2^, glomerulonephritis or nondiabetic nephropathy (with the exception of hypertensive kidney disease), autoimmune diseases, liver diseases, infection with hepatitis C virus, HIV virus, other types of diabetes such as type 1 diabetes, creatinine clearance <15 mL/min, the coexistence of other diseases with poor short-term prognosis, acute infection, pregnancy or lactation, and hospitalization in the last three months.

Patients underwent a nutritional and clinical assessment. To calculate the median daily fructose intake, a prospective diary of food ingestion for 3 days (2 week-days and one weekend day) was requested. The prospective diary consisted of a food registry that was filled prospectively for 1 week (we told the participants of the study to write down all the foods and beverages they ingested on 2 week-days and one weekend day); an example of this approach was used in another investigation [18]. Total fructose intake was estimated as the sum of the free fructose plus the fructose contained in sucrose (sucrose is composed of 50% glucose and 50% fructose). The fructose content of food was obtained from the tables of free fructose and sucrose content of the Australian Government [19] and of the United States Department of Agriculture (USDA Food Composition Databases) [20]. Two certified nutritionists (MMS and IMM), blinded to each other, calculated the daily fructose intake and the average of the 2 calculations was used for further analyses. The correlation between the 2 nutritionists was *ρ*=0.94, p<0.001.

A complete clinical evaluation was performed including past medical history, family history, and current medications. Blood pressure was measured considering the mean of two determinations after seating for at least 5 minutes [21]. Mean arterial pressure (MAP) was calculated with the traditional formula: (systolic blood pressure *∗* 0.33) + (diastolic blood pressure *∗* 0.66) [22]. Anthropometric variables were measured using standardized techniques; these included height, weight, waist and hip circumferences, and quantification of body fat using bioelectrical impedance with the Jawon scale model IOI 353 - JMW160 ®. BMI was calculated using the standard equation, weight/height^2^ (kg/m^2^).

After at least eight-hour fasting, glucose, lipid profile (total cholesterol, high-density lipoprotein (HDL)-cholesterol, triglycerides), creatinine, uric acid, alanine aminotransferase (ALT), aspartate aminotransferase (AST), and albuminuria in a 24 h urine collection were measured using an automated analyzer (Synchron CX Beckman, Fullerton CA). LDL-cholesterol (LDL) was estimated by the Friedewald equation (LDL= total cholesterol - HDL - triglycerides /5)[23]. Glycated hemoglobin (A1c) was measured using HPLC (Variant II Turbo, Biorad), certified by the National Glycohemoglobin Standardization Program (NGSP). Creatinine clearance was calculated using the CKD-EPI equation [24]. All samples were measured in the Laboratorio Central of Instituto Nacional de Ciencias Medicas y Nutricion Salvador Zubiran (http://www.innsz.mx/opencms/contenido/departamentos/labcentral/). 100% of the samples are certified by the College of American Pathologists (71893-07-01) Figure 4.

### 2.1. Ethics

The study was approved by the Comite de Etica en Investigacion del Instituto Nacional de Ciencias Medicas y Nutricion Salvador Zubiran (Ref. 939). Written informed consent was obtained from all participants before enrollment.

### 2.2. Statistics

The sample size was determined using the correlation formula. Considering a type 1 error of 5%, a 90% power, and a correlation of 0.25 between albuminuria and fructose intake, a sample of 134 patients was estimated. Numeric variables were examined for normality using the Shapiro-Wilk test. Accordingly, variables with a normal distribution are expressed as means and standard deviation, and non-normally distributed variables as medians and interquartile range.

We grouped fructose intake based on the proposal that 25 g/day (~5% of the total daily calories) or lower is the desired level [25]. Therefore, we divided the population into group 1, subjects that consumed less than 25 g day, and group 2, subjects who consumed 25 g day or more.

Comparisons between men and women were performed using independent Student's T or U-Mann-Whitney tests, as appropriate. The association between the daily fructose intake and albuminuria, uric acid, lipids, and HbA1c concentrations was evaluated with Spearman correlation coefficients. A partial correlation analysis adjusting for weight and waist circumference was also performed.

A linear regression analysis was performed to evaluate the independent association of the daily fructose intake and albuminuria. The dependent variable was albuminuria and the independent variables included in the model were the daily fructose intake, BMI, A1c, use of angiotensin-converting enzyme (ACE) inhibitors or angiotensin II receptor blockers (ARB), and the MAP.

To examine the association of fructose intake in the patients with presence of albuminuria, a subanalysis in the group of individuals with albuminuria between 30 and 3500 mg/24 h was also performed.

A two-sided p value <0.05 was considered statistically significant. Analyses were performed with the IBM SPSS Statistics version 21.0, US.

## 3. Results

**Figure 1:**
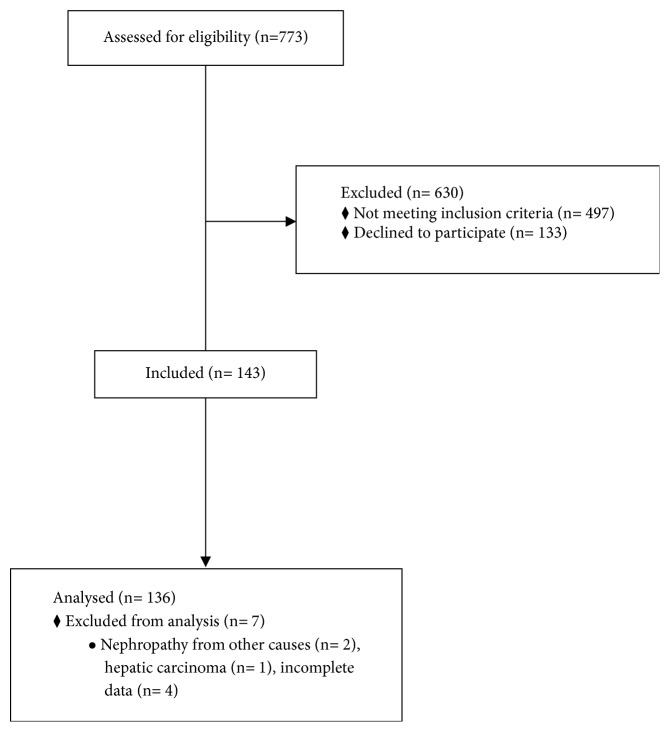
Flow chart of participants of the study.

A total of 773 subjects were assessed, of which 143 subjects were recruited; the main exclusion criteria were the presence of comorbidities such as rheumatoid arthritis, systemic lupus erythematosus, and liver disease. Seven subjects were excluded from the analysis following recruitment for other causes, leaving 136 subjects that were analyzed. The flow chart of patients included in the study is shown in Figure 1.

### 3.1. Baseline Characteristics of the Studied Population

The baseline characteristics of the studied population are summarized in Table 1. The subjects analyzed included 69 (50.7%) men and 67 (49.3%) women (the comparison of variables by sex is presented in Supplementary Table 1). The population was divided into the ones that had low fructose intake (<25 g/day) and the ones that had high fructose intake (≥ 25 g/day) (Table 1). More than 85% of participants reported a family history of T2DM in both groups (p=0.891). The mean age was similar in the low and in the high fructose intake groups (59±9.46 and 59±7.41 years, p=0.924). Current smoking was reported in 17.8% of the people with low fructose intake and in 19.6% in the group of high fructose intake (p= 0.799).

The MAP did not differ between groups (90±13.26 versus 92±11.4 mmHg, p= 0.472).

Mean BMI (29.8±4.2 versus 29.3±4.2 kg/m^2^) and waist circumference (100.4±10.8 versus 101.6±12.4 cm) were similar in low and high fructose intake groups with no statistical significance (p=0.558, p=0.574, respectively) as well as the waist-hip ratio (WHR) with 0.95±0.08 versus 0.97±0.07, p=0.084, comparing both groups. However, the body fat percentage was higher in low fructose intake compared to high fructose intake (33.8±7.6 versus 30.4±9, p <0.033).

Fasting glucose concentration was not statistically significant different between the groups (151.9±61.34 versus 160.4±69.45 mg/dL, p= 0.462). Nevertheless, HbA1c was significantly higher in the group with high fructose intake (8.6±1.61% versus 9.6±2.1%, p= 0.003). Serum creatinine was similar in both groups (1.08±93 versus 1.25±74 mg/dL, p= 0.281), but creatinine clearance was decreased in the group with high fructose intake (94.9±36.8 versus 76.5±30.98 mL/min, p=0.014). BUN and uric acid were also higher in the subjects with high fructose intake (19.6[13.6-21.3] versus 24.6[15.5-27.6] md/dL, p= 0.022 and 6.27±1.8 versus 7.2±1.5 mg/dL, p=0.012 respectively). Median albuminuria (60.8 [12.8-228.5] versus 232.2 [27.2-1273.0] mg/day, p 0.002) was also higher in subjects with high fructose intake compared to the <25 g/day fructose intake.

Both total cholesterol and LDL-cholesterol were significantly higher in the group with higher fructose intake (167.2±33.3 versus 186.5±44.4 mg/dL, p=0.005, and 91.5±28.7 versus 106.6±35.9 mg/dL, p=0.010, respectively). However, serum triglycerides (162.3[101.2-195.5] versus 162.3[101.2-195.5] mg/dL, p=0.936) and HDL-cholesterol (45.1±10.6 versus 47.5±12.7 mg/dL, p=0.230) were similar between the groups.

In relation to treatment, 77% of subjects were receiving insulin, 82% metformin, 34% an angiotensin-converting-enzyme (ACE) inhibitor, and 12% an angiotensin II receptor blocker (ARB). Insulin use was higher in the high fructose group, but for the other treatments there were no differences between the low and high fructose groups (Table 1).

In supplementary tables 1to 3 we show the subjects stratified by gender.

### 3.2. Calorie and Fructose Intake

Total energy (calorie) intake was 1585±558.4 kcal/day in the subjects with low fructose intake and 2125±588.9 kcal/day in the subjects with high fructose intake, p<0.001. The median fructose intake was 14.3±6.3 g/day in the low fructose intake group and 36.4±10.4 g/day in high fructose intake group (p<0.001). The macronutrient distribution is summarized in Table 2. The analysis by sex is shown in Table 2 of the Supplementary Materials.

### 3.3. Correlation Analysis

In Table 3, we present the correlations between albuminuria with clinical and metabolic variables in all the studied subjects (n=136). We found a positive, significant association between daily fructose intake and albuminuria (*ρ*= 0.178, p=0.038). Other variables significantly associated with albuminuria were BMI (*ρ*= 0.170, p=0.048), MAP (*ρ*= 0.280, p=0.001), A1c (*ρ*= 0.197, p=0.022), and triglycerides (*ρ*= 0.219, p=0.010).

In Table 4, we show the correlation analysis for the subjects with albuminuria between 30 and 3500 mg/day. There was a significant positive association between fructose intake and albuminuria (*ρ*= 0.238, p=0.027).

**Figure 2:**
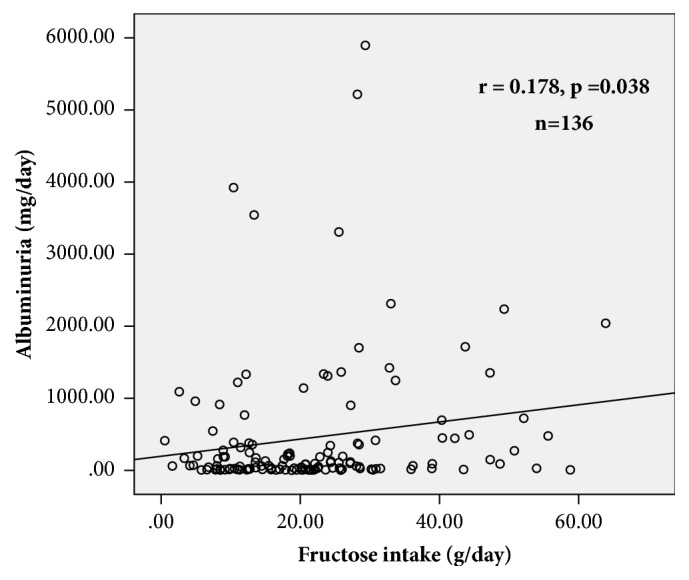
Association between fructose intake (g/day) and albuminuria (mg/day), obtained by Spearman correlation test (n=136).

**Figure 3:**
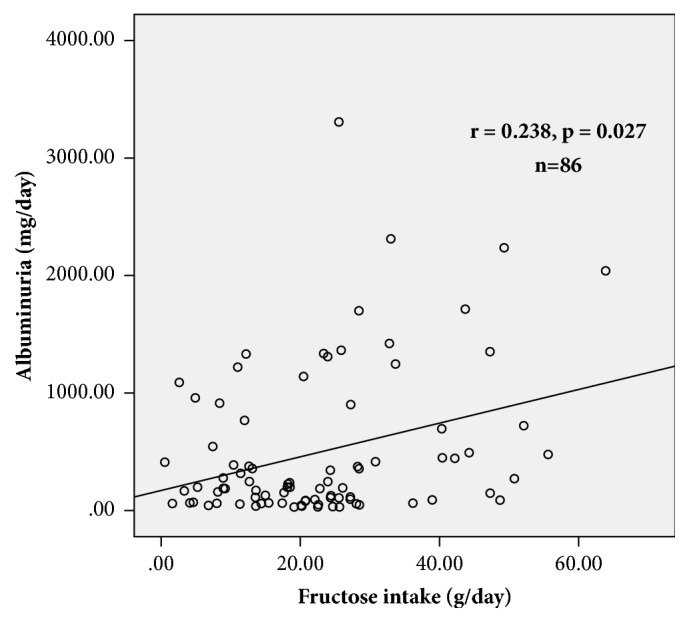
Association between fructose intake (g/day) and albuminuria (mg/day) in subjects with albuminuria between 30 to 3500 g/day, obtained by Spearman correlation test (n=86).

**Figure 4:**
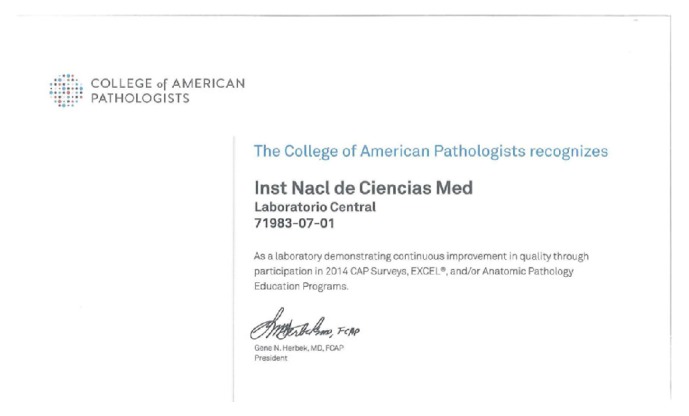
Certification of College of American Pathologists Laboratorio Central of the Instituto Nacional de Ciencias Medicas y Nutricion Salvador Zubiran.

In Figure 2 the association between fructose intake and albuminuria is illustrated in all the studied subjects (n=136). The association between fructose intake and albuminuria in subjects with albuminuria between 30 to 3500 mg/day is presented in Figure 3.

### 3.4. Multiple Linear Regression Analysis

To adjust for confounding variables, we performed a multiple linear regression analysis considering albuminuria as the dependent variable (subjects with 30 to 3500 mg/day) and fructose intake, BMI, HbA1c, use of ACE inhibitors and/or ARB, and MAP as independent variables. We found a significant and independent association between fructose intake and albuminuria (*β*= 13.96, p=0.006) (Table 5).

## 4. Discussion

This study evaluated the association between fructose intake and the following: albuminuria, glycemic control, and lipid profile, in subjects with T2DM. A significant positive association between fructose intake and albuminuria in subjects with T2DM was found. This association persisted after the adjustment for significant confounders. Fructose intake was similar between men and women, but albuminuria was higher in men than in women as previously reported [26] (Supplementary Tables 1 and 3). However, we found greater albuminuria and uric acid concentration in diabetic subjects on a high fructose diet compared to a low fructose diet.

The proposed mechanism that could explain why a high fructose diet could be associated with kidney damage is the following: Fructose is transported passively in the intestines by the GLUT5 transporter [27] and then is phosphorylated by ketohexokinase in an unregulated manner, causing hepatic ATP depletion and decreased protein synthesis, with inflammatory and prooxidant changes [28–30]. In addition, ATP depletion with nucleotide turnover favors uric acid synthesis [31] and causes the activation of the carbohydrate-responsive element-binding protein (ChREBP), a transcription factor that induces the expression of genes implicated in triglyceride synthesis [32]. Fructose also augments triglyceride synthesis by its metabolism into dihydroxyacetone phosphate and glyceraldehyde-3-phosphate, which constitutes the glycerol structural base of triglycerides [33, 34], and initiates glycolysis and production of acetyl-CoA, required for* de novo* lipogenesis and fatty acid synthesis [35]. Recently it has been demonstrated that sorbitol can be converted to fructose (fructoneogenesis) in the proximal tubule and then metabolized by the ketohexokinase, leading to ATP depletion, proinflammatory cytokine expression, and oxidative stress [36].

Some authors suggest that diabetic nephropathy could be aggravated by oxidative stress [37];in addition, both high uric acid [37] and high triglycerides [38] have been associated also with diabetic nephropathy. Therefore we think that a high fructose diet increases oxidative stress, uric acid and triglycerides, and deteriorate kidney function and increases albuminuria in subjects with T2DM.

A high fructose diet has been shown to induce renal damage in rats (3). The proposed mechanisms include synthesis of reactive oxygen species secondary to ATP depletion and uric acid generation with the production of chemokines and proinflammatory molecules [29, 30, 39]. Another study showing the deleterious effect of a high fructose diet was made in* Rhesus* monkeys that received a daily beverage containing 75 g of fructose for 12 months; those monkeys developed an increase in body fat, insulin resistance, and dyslipidemia. Furthermore, 4 monkeys (15%) developed diabetes [40]. Similar deleterious effects were observed in humans that received fructose-sweetened beverages (25% of the energy requirement) for 10 weeks compared with a group receiving glucose-sweetened beverages [13]. In an additional study, subjects who consumed a fructose-sweetened beverage had greater visceral adiposity, higher triglycerides, increased* de novo* lipogenesis, higher low-density lipoproteins (LDL) cholesterol, Apo B-100, small dense LDL, oxidized LDL, higher concentrations of glucose and insulin, and lower insulin sensitivity [41].

There is evidence supporting the beneficial effect of a low fructose diet on lipid profile, body weight, systolic and diastolic blood pressure, body fat, uric acid, triglycerides, fasting glucose, and insulin resistance in humans [42]. For example, a recent study found that obese children who had their fructose intake reduced showed a reduction in liver fat and an improvement in insulin kinetics [43]. Furthermore, in patients with nondiabetic chronic kidney disease stages 2 and 3, a low fructose diet exerted a decrease in uric acid, arterial blood pressure, and insulin concentration [44].

In a subanalysis of the Nurses' Health Study using semiquantitative food frequency questionnaires to estimate diet composition, a higher dietary intake of animal fat and two or more servings per week of red meat were associated with an increased risk of microalbuminuria, and after adjustment for other nutrients individually associated with eGFR decline >30%, only the highest quartile of sodium intake remained directly associated (OR: 1.52; 95% CI: 1.10 to 2.09), whereas *β*-carotene appeared protective (OR: 0.62, 95% CI: 0.43 to 0.89) [45]. Although we did not measure animal fat, red meat, or sodium intake, subjects with a high fructose intake (≥25 g/day) ingested less % of protein (16.9±3.2 % versus 19.3±4.7%, p = 0.002); the amount of %fat of the diet was similar between both groups: 36.5±9.6 versus 34±9.2, p 0.152.

The limitations of this study are that the exact content of free fructose in some processed foods could not be determined. For this reason, the amount of fructose was estimated dividing the sucrose content by 2. This may underestimate the real daily intake of fructose. Despite this, a significant positive association between fructose intake and albuminuria was found. Another limitation is that the cross-sectional design of the study does not permit inferring any causality between fructose intake and albuminuria, so additional prospective studies are needed to assess if low fructose diet could decrease albuminuria.

Another important limitation is that since all subjects consumed different types of fructose-containing foods, other nutrient factors contained in the diet that improve diabetes or insulin resistance (e.g., magnesium [46] or chromium[47]) were not considered and could be potential confounders of the study.

Another confounder factor that should be addressed in further longitudinal studies is that in our study subjects in the high fructose intake group also were the ones that had a higher intake in calories.

The strengths of this study are that we had a sufficient sample size to obtain statistically significant results; subjects underwent a comprehensive clinical and biochemical evaluation, in whom a prospective registry of food intake (the gold standard to calculate nutrient intake) during three days was performed to quantify the fructose intake. To our knowledge this is the first time fructose consumption was measured in patients in the Instituto Nacional de Ciencias Medicas y Nutricion Salvador Zubiran.

Further studies evaluating the effect of low fructose diets in reducing albuminuria and improving renal function in selected individuals are needed to warrant the implementation of preventive strategies. In summary, in subjects with T2DM we found a positive and independent association between fructose intake and albuminuria.

## Figures and Tables

**Table 1:** Baseline characteristics of the subjects included in the study classified by fructose intake (n = 136).

**Variable**	**All**	**Low fructose intake (**<**25 g/day)**	**High fructose intake (**≥**25 g/day)**	**p**
	**n** **= 136**	**n** **= 90 **	**n** **= 46**	
Age, years	59±8.79	59±9.46	59±7.41	0.924
Family history of T2DM	119, 87.5%	79, 87.8%	40, 87%	0.891
Diabetes duration, years	16.5±7.8	16±8	17±7	0.443
Current smoking	25, 18.4%	16, 17.8%	9, 19.6%	0.799
MAP, mmHg	90.9±12.6	90±13.26	92±11.4	0.472
BMI, kg/m^2^	29.6±4.2	29.8±4.2	29.3±4.2	0.558
Waist circumference, cm	100.8±11.3	100.4±10.8	101.6±12.4	0.574
WHR	0.95±.08	0.95±0.08	0.97±0.07	0.084
Body fat, %	32.7±8.2	33.8±7.6	30.4±9	0.033
Glucose, mg/dL	154.8±64.0	151.9±61.34	160.4±69.45	0.462
A1c, %	8.9±1.8	8.6±1.61	9.6±2.1	0.003
Creatinine, mg/dL	0.95[0.74-1.2]	1.08±0.93	1.25±0.74	0.281
BUN, mg/dL	17.0 [14-23.8]	19.6[13.6-21.3]	24.6[15.5-27.6]	0.022
Uric acid, mg/dL	6.6±1.8	6.27±1.8	7.2±1.5	0.012
Total cholesterol, mg/dL	173±38.4	167.2±33.3	186.5±44.4	0.005
Triglycerides, mg/dL	142.5 [104.2- 189.7]	162.3[101.2-195.5]	166.6[112.5-180.7]	0.936
LDL-cholesterol, mg/dL	96.7±32.0	91.5±28.7	106.6±35.9	0.010
HDL-cholesterol, mg/dL	45.9±11.4	45.1±10.6	47.5±12.7	0.230
Creatinine clearance, mL/min	88.3±35.8	94.9±36.8	76.5±30.9	0.014
Albuminuria, mg/day	87.3 [16.4-385.5]	60.8 [12.8-228.5]	232.2 [27.2 1273.0]	0.002
Insulin use	106, 77.9%	64, 71.1%	42, 91.3%	0.007
Insulin dose, U/kg	40 [24-53.2]	43.6[24.2-56.7]	40.1[22.7-51.7]	0.747
Metformin use	112, 82.4%	78, 86.7%	34, 73.9%	0.065
Sulfonylurea use	14, 10.3%	11, 12.2%	3, 6.5%	0.301
DPP-4 inhibitor use	10, 7.4%	6, 6.7%	4, 8.7%	0.668
ACE inhibitor use	47, 34.6%	31, 34.4%	16, 34.8%	0.969
ARB use	17, 12.5%	9, 10%	8, 17.4%	0.218
Aspirin use	69, 50.7%	44, 48.9%	25, 54.3%	0.547
Statin use	74, 54.4%	51, 56.7%	23, 50%	0.460
Fibrate use	34, 25%	26, 28.9%	8, 17.4%	0.143

Data is expressed as mean ± standard deviation, median [interquartile range], or numbers (percentage). P values obtained according to student T, U Mann-Whitney, or chi-squared tests as appropriate. MAP: mean arterial pressure, BMI: body mass index, WHR: waist to hip ratio, A1c: glycated hemoglobin, DPP-4: Dipeptidyl peptidase-4, ARB: angiotensin receptor blockers, ACE: angiotensin converting enzyme

**Table 2:** Calorie and fructose intake in low and high fructose intake groups.

	**All**	**Low fructose intake (<25 g/day)**	**High fructose intake (≥25 g/day)**	**p**
	**n=136**	**n= 90 **	**n= 46**	
Total Calories kcal/day	1768±622.1	1585±558.4	2125±588.9	<0.001
Carbohydrates, %	45.6±11.9	43.9±12.3	48.8±10.5	0.022
Proteins, %	18.5±4.4	19.3±4.7	16.9±3.2	0.002
Fat, %	35.7±9.5	36.5±9.6	34±9.2	0.152
Fructose, %	4.9 [2.9-6.6]	4.0[2.3-5.1]	6.6[5.4-8.2]	<0.001
Fructose intake, g/day	14.5 [9.4-21.9]	13.5[9.1-20.2]	32.1[28.1-43.8]	<0.001

Data expressed as mean ± standard deviation or median [interquartile range]. P values obtained according to student T or U Mann-Whitney tests, as appropriate.

**Table 3:** Correlation analyses between albuminuria and creatinine clearance with clinical and metabolic variables (n= 136).

**Variable**	**Albuminuria, ** **mg/day** **rho**	**p**	**CKD-EPI, ** **mL/min** **rho**	**P**	**Measured creatinine clearance, ** **mL/min** **rho**	**p**
Fructose intake, g/day	0.178	0.038	-0.106	0.221	-0.032	0.749
Smoking, pack-years	0.191	0.394	-0.173	0.441	-0.086	0.719
BMI, kg/m^2^	0.170	0.048	-0.051	0.558	0.073	0.473
MAP, mmHg	0.280	0.001	-0.055	0.526	-0.097	0.344
A1c, %	0.197	0.022	-0.109	0.207	-0.069	0.503
Triglycerides, mg/dL	0.219	0.010	-0.156	0.069	-0.102	0.319
Total cholesterol, mg/dL	0.138	0.110	0.114	0.188	-0.101	0.320
HDL-c, mg/dL	-0.131	0.127	0.048	0.582	0.073	0.472
LDL-c, mg/dL	0.084	0.334	-0.018	0.834	-0.083	0.418

p values obtained by Spearman correlation analyses. BMI: body mass index, MAP: mean arterial pressure, A1c: glycated hemoglobin

**Table 4:** Correlation analyses in subjects with albuminuria between 30 and 3500 g/day (n=86).

**Variable**	**Albuminuria, mg/day** **rho**	**p**	**CKD-EPI, mL/min** **rho**	**p**	**Measured creatinine clearance, mL/min** **rho**	**p**
Fructose intake, g/day	0.238	0.027	0.091	0.404	-0.322	0.013
Smoking, pack-years	-0.370	0.175	0.072	0.800	0.154	0.616
BMI, kg/m^2^	0.021	0.851	-0.041	0.707	0.164	0.215
MAP, mmHg	0.075	0.491	0.082	0.455	0.63	0.638
A1c, %	0.046	0.675	.029	0.788	-0.042	0.755
Triglycerides, mg/dL	0.092	0.398	-0.116	0.288	0.117	0.378
Total cholesterol, mg/dL	0.082	0.455	-0.028	0.797	-0.077	0.562
HDL-c, mg/dL	-0.148	0.174	0.051	0.638	-0.022	0.866
LDL-c, mg/dL	0.054	0.625	0.049	0.653	0.012	0.928

p values obtained by Spearman correlation analyses. BMI: body mass index, MAP: mean arterial pressure, A1c: glycated hemoglobin

**Table 5:** Multiple linear regression analysis of variables associated with albuminuria in the subjects included in the study with albuminuria between 30 and 3500 mg/day (n = 86).

**Variable**	β	**t**	**p**
Constant	-857.47	-1.20	0.23
Fructose intake, g/day	13.96	2.82	0.006
BMI, kg/m^2^	19.30	1.24	0.219
A1c, %	23.84	0.585	0.560
ACE inhibitor and/or ARB use	121.32	0.913	0.364
MAP, mmHg	1.842	0.349	0.728

Parameters of the model: r=0.36, r^2^=0.13, F=2.44, and p=0.041. BMI: body mass index, A1c: glycated hemoglobin, ACE: angiotensin converting enzyme, ARB: angiotensin receptor blocker, MAP: mean arterial pressure.

## Data Availability

All the authors agree to share the database. Dataset is available from the corresponding author at gomezperezfco@gmail.com.

## Supplementary Materials

Supplementary Materials Supplementary Table 1: baseline characteristics of the subjects included in the study (n = 136). Supplementary Table 2: calorie and fructose intake by sex. Supplementary Table 3: albuminuria, fructose intake, and creatinine clearance stratified by sex.
