# Supplementary material for: A Higher Fructose Intake Is Associated with Greater Albuminuria in Subjects with Type 2 Diabetes Mellitus
Source: Int J Nephrol. 2018 Oct 17;2018:5459439. doi: 10.1155/2018/5459439 (PMC6207863; doi:10.1155/2018/5459439)
Supplement: Supplementary Materials — Supplementary Table 1: baseline characteristics of the subjects included in the study (n = 136). Supplementary Table 2: calorie and fructose intake by sex. Supplementary Table 3: albuminuria, fructose intake, and creatinine clearance stratified by sex. [file 5459439.f1.docx]

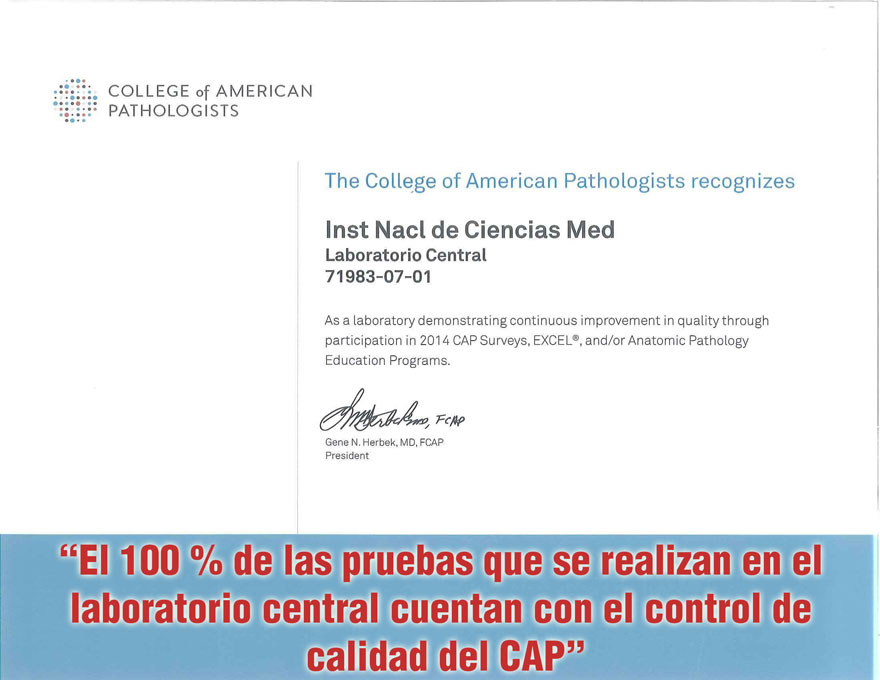


**Supplementary Materials**

**Supplementary table 1.** Baseline characteristics of the subjects included in the study (n = 136)

| **Variable** | **All**  **n= 136** | **Men**  **n= 69** | **Women**  **n= 67** | **p** |
| --- | --- | --- | --- | --- |
| Age, years | 59±8.79 | 59.6±8.0 | 58.4±9.5 | 0.399 |
| Family history of T2DM | 119, 87.5% | 59, 85.5% | 60, 89.6% | 0.476 |
| Diabetes duration, years | 16.5±7.8 | 15.8±7.4 | 17.2±8.2 | 0.275 |
| Current smoking | 25, 18.4% | 18, 26.1% | 7, 10% | 0.019 |
| MAP, mmHg | 90.9±12.6 | 93.1±12.9 | 88.7±12.0 | 0.045 |
| BMI, kg/m^2^ | 29.6±4.2 | 29.0±4.2 | 30.2±4.1 | 0.100 |
| Waist circumference, cm | 100.8±11.3 | 102.7±12.2 | 98.9±10.0 | 0.053 |
| WHR ratio | 0.95±.08 | 0.99±.08 | 0.92±.066 | <0.0001 |
| Body fat, % | 32.7±8.2 | 27.6±6.7 | 37.6±6.3 | <0.0001 |
| Glucose, mg/dL | 154.8±64.0 | 156.7±66 | 152.7±62.4 | 0.718 |
| A1c, % | 8.9±1.8 | 9.0±2.0 | 8.9±1.5 | 0.627 |
| Creatinine, mg/dL | 0.95[0.74-1.2] | 1.0 [0.85-1.3] | 0.79 [0.65-1.05] | <0.0001 |
| BUN, mg/dL | 17.0 [14-23.8] | 18.6 [14.5-26.3] | 16.5 [13.1-23.1] | 0.067 |
| Uric acid, mg/dL | 6.6±1.8 | 7.0±1.9 | 6.0±1.4 | 0.006 |
| Total cholesterol, mg/dL | 173±38.4 | 171.6±38.0 | 175.9±39.0 | 0.512 |
| Triglycerides, mg/dL | 142.5 [104.2- 189.7] | 141 [108.0-189.5] | 144 [100.0-191.0] | 0.901 |
| LDL-cholesterol, mg/dL | 96.7±32.0 | 95.6±30.1 | 97.9±34.1 | 0.686 |
| HDL-cholesterol, mg/dL | 45.9±11.4 | 42.7±9.9 | 49.2±12.0 | 0.001 |
| Creatinine clearance, mL/min | 88.3±35.8 | 72.4±25.4 | 77.4±26.5 | 0.266 |
| Albuminuria, mg/day | 87.3 [16.4-385.5] | 225.7 [30.9-620.6] | 43.7 [11.0-167.4] | <0.0001 |
| Insulin use | 106, 77.9% | 53, 76.8 % | 53, 79.1 % | 0.747 |
| Insulin dose, U/kg | 40 [24-53.2] | 0.50 [0.29-0.66] | 0.56 [0.35-0.76] | 0.21 |
| Metformin use | 112, 82.4% | 54, 78.3% | 58, 86.6% | 0.204 |
| Sulfonylurea use | 14, 10.3% | 9, 13% | 5, 7.5% | 0.284 |
| DPP4 inhibitor use | 10, 7.4% | 6, 8.7% | 4, 6% | 0.543 |
| ACE inhibitor use | 47, 34.6% | 24, 34.8 % | 23, 34.3 % | 0.956 |
| ARB use | 17, 12.5% | 12, 17.4 % | 5, 7.5 % | 0.080 |
| Aspirin use | 69, 50.7% | 40, 58 % | 29, 43.3 % | 0.087 |
| Statin use | 74, 54.4% | 32, 46.4 % | 42, 62.7 % | 0.056 |
| Fibrate use | 34, 25% | 18, 26.1 % | 16, 23.9 % | 0.844 |

Data expressed as mean ± standard deviation, median [interquartile range] or numbers (percentage). P values obtained according to student T, U Mann-Whitney, or chi-squared tests as appropriate. MAP: mean arterial pressure, BMI: body mass index, WHR: waist to hip ratio, A1c: glycated hemoglobin, ARB: angiotensin receptor blockers, ACE: angiotensin converting enzyme

**Supplementary table 2**. Calorie and fructose intake by sex

|  | **All**  **n=136** | **Men**  **n= 69** | **Women**  **n= 67** | **p** |
| --- | --- | --- | --- | --- |
| Total Calories kcal/day | 1768±622.1 | 1934.7±600.8 | 1597.1±600.8 | 0.001 |
| Carbohydrates, % | 45.6±11.9 | 44.7±12.4 | 46.4±11.4 | 0.409 |
| Proteins, % | 18.5±4.4 | 18.3±4.5 | 18.7±4.4 | 0.547 |
| Fat, % | 35.7±9.5 | 36.7±9.9 | 34.6±9.1 | 0.194 |
| Fructose, % | 4.9 [2.9-6.6] | 5.0 [2.6.-7.1] | 4.8 [3.8-6.3] | 0.691 |
| Fructose intake, g/day | 14.5 [9.4-21.9] | 22.6 [12.6-28.9] | 18.5 [10.4-26] | 0.059 |

Data expressed as mean ± standard deviation or median [interquartile range]. P values obtained according to student T or U Mann-Whitney tests, as appropriate

**Supplementary table 3**

Albuminuria, Fructose intake, and creatinine clearance stratified by sex

|  | **men** | **n=69** | **p** | **women** | **n=67** | **p** |
| --- | --- | --- | --- | --- | --- | --- |
| **Variable** | Low fructose  (<25 g/day) | High fructose  (≥25 g/day) |  | Low fructose  (<25 g/day) | High fructose  (≥25 g/day) |  |
| **Albuminuria**  **(mg/day)** | 152.8 (22.7 to 350.2) | 446.6 (89.2 to 1407.4) | 0.002 | 43.7 (8.8 to 147.2) | 44.4 (15.1 to 267.8) | 0.39 |
| **Fructose intake (g/day)** | 13.3 (10.4 to 20.9) | 32.1 (28.2 to 48.34) | <0.001 | 14.5 (8.2 to 20.2) | 32.2 (27.1 to 43.5) | <0.001 |
| **Creatinine clearance**  **(ml/min)** | 90.7 (78.9 to 121.0) | 63.7 (36.5 to 92.7) | 0.004 | 94.3 (64.6 to 122.5) | 96.5 (82.3 to 106.1) | 0.89 |

Data expressed as median (interquartile range). P values obtained by U Mann-Whitney tests, as appropriate
